# Therapeutic plasma exchange in autoimmune diseases: a retrospective study in a tertiary pediatric hospital in Mexico

**DOI:** 10.3389/fped.2025.1680460

**Published:** 2025-10-24

**Authors:** Héctor Menchaca-Aguayo, Deshire Alpízar-Rodríguez, Candy León-Rodríguez, Rita Gutiérrez-Hernández, Enrique Faugier-Fuentes

**Affiliations:** ^1^ Pediatric Rheumatology Department, Hospital Infantil de México Federico Gómez, Mexico City, Mexico; ^2^ Division of Rheumatology, National Institute of Rehabilitation “Luis Guillermo Ibarra Ibarra”, Mexico City, Mexico

**Keywords:** therapeutic plasma exchange, autoimmune diseases, systemic lupus erythematosus, pediatrics, sepsis, mortality

## Abstract

**Objective:**

To describe the use and mortality-associated factors of therapeutic plasma exchange (TPE) in children with autoimmune diseases at a tertiary pediatric hospital in Mexico.

**Methods:**

Retrospective cross-sectional study including patients under 18 years old with autoimmune diseases who underwent TPE between 2022 and 2025 at the Hospital Infantil de México Federico Gómez. Demographic, clinical, therapeutic, and laboratory data were analyzed. Clinical response and mortality-associated factors were assessed.

**Results:**

Forty-one patients were included (68% female; median age: 12 years). The most frequent indications for TPE were systemic lupus erythematosus (SLE) (56.1%) and autoimmune encephalitis (14.6%). Other diagnoses included macrophage activation syndrome (MAS) (*n* = 3), overlap syndromes (*n* = 3), juvenile dermatomyositis (*n* = 2), and single cases of ANCA-associated vasculitis, Takayasu arteritis, antiphospholipid syndrome (APS), and Kawasaki disease. Complete or partial remission was achieved in 85.4% of cases, with variable responses depending on the underlying condition. TPE was well tolerated, with few adverse events. Overall mortality was 14.6% (*n* = 6), all due to sepsis (*p* < 0.001). Deceased patients had higher pre-TPE levels of CRP, procalcitonin, and ferritin, though not statistically significant. Longer disease duration was significantly associated with increased mortality in both univariable (OR 1.05, 95% CI 1.01–1.10; *p* = 0.04) and multivariable analysis adjusted for age, sex, and SLE diagnosis (OR 1.06; 95% CI 1.01–1.14; *p* = 0.04). Among SLE patients without sepsis, MEX-SLEDAI scores improved significantly.

**Conclusion:**

TPE was safe and beneficial in children with severe autoimmune diseases. Mortality was related to disease duration rather than the procedure itself.

## Introduction

Therapeutic plasma exchange (TPE) involves the automated and non-selective removal of plasma and its replacement with a fluid, typically including albumin or blood components (1). The main mechanisms of action of TPE include the removal of circulating autoantibodies, immune complexes, complement components, cytokines, and adhesion molecules, as well as the sensitization of antibody-producing cells to immunosuppressive drugs (2). Autoimmune diseases are heterogeneous. While the production of autoantibodies is central in the pathogenesis of several disorders (e.g., systemic lupus erythematosus, myasthenia gravis), other autoimmune conditions are not antibody-mediated but rather driven by *T*-cell responses or dysregulated innate immunity (e.g., multiple sclerosis, juvenile idiopathic arthritis) (3). These conditions arise from the interaction between genetic and environmental factors that influence cell development, cytokine production, and autoantibody formation, leading to organ- or system-specific inflammation (4). The use of TPE in these diseases is well supported, particularly in acute and critical phases when immunosuppressive therapies may take time to become effective and timely intervention is essential (5). The use of TPE has increased in recent years due to growing evidence supporting its utility in various autoimmune diseases (6–9). However, studies on TPE use in children are limited, and adult data cannot be directly extrapolated. The management of TPE in pediatric patients requires adapted protocols that consider challenges such as metabolic immaturity, lack of cooperation, difficulties in toxicity assessment, and management of the extracorporeal circuit due to small body size (10). The global burden of autoimmune diseases in childhood is increasing, although most epidemiological data come from cohorts in developed countries and may not reflect the situation in other regions (11), but the literature on TPE use in children is scarce. This information gap in the region underscores the need for local studies that provide relevant data to the medical community in similar settings. The objective of our study was to describe the use of TPE in children with autoimmune diseases treated at the Hospital Infantil de México Federico Gómez, and to describe demographic characteristics of patients, main indications for TPE, observed side effects and associated mortality rate.

## Materials and methods

A cross-sectional, retrospective study was conducted at the Hospital Infantil de México Federico Gómez, a national pediatric referral center. All patients under 18 years of age with a diagnosis of autoimmune disease who underwent TPE between 2022 and 2025 were included. All included patients presented with moderate to high disease activity and/or refractoriness to conventional treatment with corticosteroids or intravenous immunoglobulin. The study protocol was approved by the local ethics committee (approval number HIM/SR/2025/004). As this study involved the review of de-identified data, written informed consent was not required. Information was obtained from medical records and collected using a standardized form that included the following variables: sex, age, diagnosis, presence of sepsis, classification according to the American Society for Apheresis (ASFA) (12), number of TPE sessions performed, and type of replacement fluid used. Notably, ASFA classification was applied retrospectively for analytical purposes and did not guide the initial clinical decision to perform TPE. Briefly, ASFA categories indicate the strength of evidence for TPE in each disorder: Category I (first-line therapy), Category II (second-line therapy), Category III (role not well established), and Category IV (ineffective or potentially harmful). TPE was scheduled on alternating days, with an average of five sessions per patient. Procedures were performed using the Spectra Optia® apheresis system through continuous flow centrifugation and a central venous catheter. The exchanged volume was equivalent to 1.5 times the estimated total blood volume. The replacement fluid used was predominantly 5% albumin, and citrate was used as the anticoagulant. The complete technical protocol is described in Supplementary Material S1. The total number of sessions per patient was determined based on the diagnosis, clinical response to treatment, and occurrence of adverse events. Clinical response was evaluated during the first week following TPE using the criteria proposed by Bai et al. (13), which define complete remission as normalization of laboratory tests and absence of clinical symptoms, partial remission as an improvement of at least 50% in laboratory parameters compared to baseline without the appearance of new symptoms, and persistence or worsening as continued or worsening abnormalities in laboratory findings and/or clinical symptoms. The assessment of clinical response was performed by the treating team. Deaths that occurred during the hospitalization in which TPE was administered were recorded.

### Statistical analysis

Quantitative variables were summarized using median and interquartile range, while qualitative variables were described using proportions. Chi-square or Fisher's exact tests were used to compare categorical variables between groups. Depending on the distribution of continuous data, Student's *t*-test or the Kruskal–Wallis test was applied, followed by Dunn's test for multiple comparisons. Bonferroni adjustment was used for multiple testing. To identify factors associated with mortality, univariable and multivariable logistic regression analyses were performed. In univariable logistic regression, if a variable perfectly predicted the outcome and the Chi-square test yielded a *p*-value < 0.001, Firth's penalized logistic regression was applied. For the multivariable model, backward elimination was used based on clinical and/or statistical significance. The Hosmer-Lemeshow test was conducted to assess model fit and calibration. A *p*-value < 0.05 was considered statistically significant. Statistical analysis was performed using STATA version 14.0 (Stata Corp LP, College Station, TX, USA).

## Results

A total of 44 patients with autoimmune diseases who received TPE were initially included; however, three were excluded due to incomplete information in the medical records. Therefore, the final analysis included 41 patients. The median age was 12 years (IQR 8–14), with a predominance of females (68%). The median disease duration prior to initiating TPE was 2 months (IQR 1–12). The most frequent diagnosis was SLE (56.1%), followed by autoimmune encephalitis (14.6%). Three patients presented with macrophage activation syndrome, one associated with autoimmune panuveitis and two with systemic juvenile idiopathic arthritis (sJIA) (Table 1). Thirty-nine percent of the patients had a concurrent diagnosis of sepsis. A median of five TPE sessions was performed per patient (IQR 5–5). The most commonly used replacement fluid was 5% albumin (92.7%), while three patients received fresh frozen plasma (FFP), selected based on the presence of coagulopathy and sepsis, conditions that require plasma factor replacement. According to the ASFA classification, the most frequent therapeutic indication corresponded to category III (48.8%), followed by category II (26.8%) and category I (24.4%) (Table 1). Adverse events occurred in only five patients, with palpitations being the most common. Specifically, Patient 1 experienced symptoms of hypocalcemia; Patient 2 experienced palpitations and hypotension; Patient 3 experienced palpitations, dizziness, and nausea; Patient 4 developed coagulopathy; and Patient 5 experienced hypotension. Before starting TPE, 82.5% of patients had hemodynamic compromise, followed by respiratory (74.2%) and neurological involvement (70.4%). Regarding treatment response, 41.5% of patients achieved complete remission, 43.9% had partial remission, and 14.6% showed persistence or worsening of the disease. Specifically, 52.9% of patients with SLE and 29.4% of those with autoimmune encephalitis achieved complete remission. Partial remission was predominantly observed in patients with SLE (61.1%), while the remainder exhibited persistent or worsening clinical features (Table 2). MAS was documented in three patients, two with sJIA and one with autoimmune panuveitis. All three presented with severe clinical courses, including multiorgan dysfunction and the need for intensive care support. None achieved complete remission, and one patient died. Overlap syndromes were identified in three patients (SLE with systemic sclerosis, juvenile dermatomyositis with systemic sclerosis, and SLE with JIA), showing variable responses: two achieved complete remission and one partial remission. Patients with ANCA-associated vasculitis, Takayasu arteritis, APS, and Kawasaki disease were single cases; all achieved partial remission except for the ANCA-associated vasculitis case, who achieved complete remission. Juvenile dermatomyositis was diagnosed in two patients—one had partial remission and the other showed persistent disease. Overall mortality was 14.6% (6 deaths), none of which were attributed to the procedure (Table 3; Supplementary Material S2). Five of the six deceased patients (83.3%) had a diagnosis of SLE. All patients who died had sepsis (*p* < 0.001, by Fisher's exact test). In univariable analysis, since the variable sepsis perfectly predicted treatment failure, Firth's penalized logistic regression was applied (OR 3.5; 95% CI: 0.5–6.4) (Table 3). Regarding inflammatory biomarkers, deceased patients showed lower ESR levels (median 0 mm/h, IQR 0–4 vs. 22 mm/h, IQR 20–30) and higher levels of CRP (7.0 vs. 2.3 mg/dl), procalcitonin (3.2 vs. 0.3 ng/mL), and ferritin (5,520 vs. 1,130 ng/mL), although none of these differences reached statistical significance (Table 3). In univariable analysis, longer disease duration was significantly associated with mortality (OR 1.05; 95% CI 1.01–1.10, *p* = 0.046). The association persisted in the multivariable analysis, adjusted by age, sex and lupus diagnosis (OR 1.06; CI 95%: 1.01–1.14; *p* = 0.04). The Hosmer-Lemeshow test showed good model fit (*p* = 0.40). In SLE patients without sepsis (*n* = 11), disease activity was assessed before and after TPE using the MEX-SLEDAI score (Supplementary Material S2). The median pre-TPE SLEDAI score was 10 (IQR 9–11), and the median post-TPE score was 4 (IQR 2–6). Disease activity before and after the procedure was compared using the Wilcoxon signed-rank test, showing a statistically significant reduction in the MEX-SLEDAI score [median pre 10 [IQR 9–11] vs. median post 4 [IQR 2–6], *p* = 0.0030].

**Table 1:** Clinical, diagnostic, and therapeutic characteristics of patients undergoing therapeutic plasma exchange, stratified by sex (*n* = 41).

Characteristic	Total	Male (*n* = 13)	Female (*n* = 28)	*p*-value
Age, years, median (IQR)	12 (8–14)	10 (8–12)	12 (8.5–14)	0.07
Disease duration, months, median (IQR)	2 (1–12)	2 (1–9)	2.5 (1–21.5)	0.37
Diagnosis, *n* (%)				
SLE	23 (56.1)	4 (30.8)	19 (67.9)	0.014
Autoimmune encephalitis	6 (14.6)	3 (23.1)	3 (10.7)	
Overlap syndrome^a^	3 (7.3)	0 (0.0)	3 (10.7)	
Macrophage activation syndrome (MAS)	3 (7.3)	3 (23.1)	0 (0.0)	
Juvenile dermatomyositis	2 (4.9)	1 (7.7)	1 (3.6)	
ANCA-associated vasculitis	1 (2.4)	0 (0)	1 (3.6)	
Antiphospholipid antibody syndrome (APS)	1 (2.4)	0 (0)	1 (3.6)	
Takayasu arteritis	1 (2.4)	1 (7.7)	0 (0)	
Kawasaki disease	1 (2.4)	1 (7.7)	1 (3.6)	
Sepsis, *n* (%)	16 (39.0)	5 (38.5)	11 (39.3)	1.00
Total sessions, median (IQR)	5 (5–5)	5 (5–5)	5 (5–5)	0.25
Replacement fluid albumin, *n* (%)	38 (92.7)	11 (84.6)	27 (96.4)	0.23
ASFA category, *n* (%)				
Category I	10 (24.4)	3 (23.1)	7 (25.0)	0.47
Category II	11 (26.8)	2 (15.4)	9 (32.1)	
Category III	20 (48.8)	8 (61.5)	12 (42.9)	
Patients with adverse events (AEs)	5 (12.2)	2 (15.4)	3 (10.7)	0.64
Respiratory involvement	23 (74.2)	7 (63.6)	16 (80.0)	0.40
Neurological involvement	19 (70.4)	6 (60.0)	13 (76.5)	0.41
Hemodynamic involvement	33 (82.5)	9 (69.2)	24 (88.9)	0.18
Clinical response to TPE				
Complete remission	17 (41.5)	3 (23.1)	14 (50.0)	0.25
Partial remission	18 (43.9)	7 (53.9)	11 (39.3)	
Persistence/worsening	6 (14.6)	3 (23.1)	3 (10.7)	

*P*-value calculated by Chi-square test for categorical variables and the Kruskal–Wallis test for continuous variables. Dunn's test with Bonferroni correction was used for multiple comparisons.

^a^ SLE + systemic sclerosis (*n* = 1), juvenile dermatomyositis + systemic sclerosis (*n* = 1), and SLE + JIA (*n* = 1). IQR, interquartile range.

**Table 2:** Distribution of diagnoses according to clinical response one week after therapeutic plasma exchange (TPE).

Diagnoses *n* (%)	Complete remission (*n* = 17)	Partial remission (*n* = 18)	Persistence worsening (*n* = 6)	*p*-value
SLE	9 (52.9)	11 (61.1)	3 (50.0)	0.11
Autoimmune encephalitis	5 (29.4)	1 (5.6)	0 (0.0)	
Overlap syndrome^a^	2 (11.8)	1 (5.6)	0 (0.0)	
MAS	0 (0.0)	2 (11.1)	1 (16.7)	
Juvenile dermatomyositis	0 (0.0)	1 (5.6)	1 (16.7)	
ANCA-associated vasculitis	1 (5.9)	0 (0.0)	0 (0.0)	
Antiphospholipid antibody syndrome (APS)	0 (0.0)	0 (0.0)	1 (16.7)	
Takayasu arteritis	0 (0.0)	1 (5.6)	0 (0.0)	
Kawasaki disease	0 (0.0)	1 (5.6)	0 (0.0)	

*P*-values were calculated using Fisher's exact test.

^a^SLE + systemic sclerosis (*n* = 1), juvenile dermatomyositis + systemic sclerosis (*n* = 1), and SLE + JIA (*n* = 1).

**Table 3:** Univariable comparison of clinical characteristics, diagnoses, and laboratory findings between deceased and surviving patients following therapeutic plasma exchange (TPE).

Variables	Non-survivors (*n* = 6, 14.6%)	Survivors (*n* = 35, 85.4%)	OR (IC 95%)
Sex, female, *n* (%)	3 (50.0)	25 (71.4)	0.4 (0.1–2.3)
Age, years, median (IQR)	13 (12–15)	11 (8–14)	1.2 (0.9–1.7)
Disease duration, months, median (IQR)	11 (2–60)	2 (1–10)	1.04 (1.0–1.1)
Diagnosis, *n* (%)			
SLE	5 (83.3)	18 (51.4)	0.9 (0.7–1.2)
Autoimmune encephalitis	0 (0.0)	6 (17.1)	
Overlap syndrome	0 (0.0)	3 (8.6)	
MAS	1 (16.7)	2 (5.7)	
Juvenile dermatomyositis	0 (0.0)	2 (5.7)	
ANCA-associated vasculitis	0 (0.0)	1 (2.9)	
APS	0 (0.0)	1 (2.9)	
Takayasu arteritis	0 (0.0)	1 (2.9)	
Kawasaki disease	0 (0.0)	1 (2.9)	
Sepsis, *n* (%)	6 (100.0)	10 (28.6)	3.5 (0.5–6.4)^e^
Erythrocyte sedimentation rate (ESR), median (IQR) (mm/hr)^a^	0 (0–4)	22 (20–30)	0.8 (0.6–1.1)
CRP, median (IQR) (mg/dl)^b^	7.0 (4.0–7.5)	2.3 (0.3–3.5)	1.1 (0.9–1.3)
Procalcitonin, *n* (%)^c^ (ng/mL)	3.2 (1.7–3.7)	0.3 (0.1–1.1)	1.2 (0.7–2.0)
Ferritin, median (IQR)^d^ (ng/mL)	5,520 (2,640–30,100)	1,130 (369–2,770)	1.0 (0.9–1.0)
Leukocytes, median (IQR) (/μl)	9,900 (6,900–15,000)	8,900 (4,900–12,100)	1.0 (0.9–1.0)
LDH, median (IQR) (U/L)	397 (222–530)	253 (183–475)	1.0 (0.9–1.0)
Fibrinogen, median (IQR) (mg/dl)	218 (119–304)	212 (123–337)	0.9 (0.9–1.0)

OR, odds ratio.

^a^ 16 patients.

^b^ 25 patients.

^c^ 17 patients.

^d^ 14 patients.

^e^ Firth's logistic regression.

## Discussion

**Figure 1:**
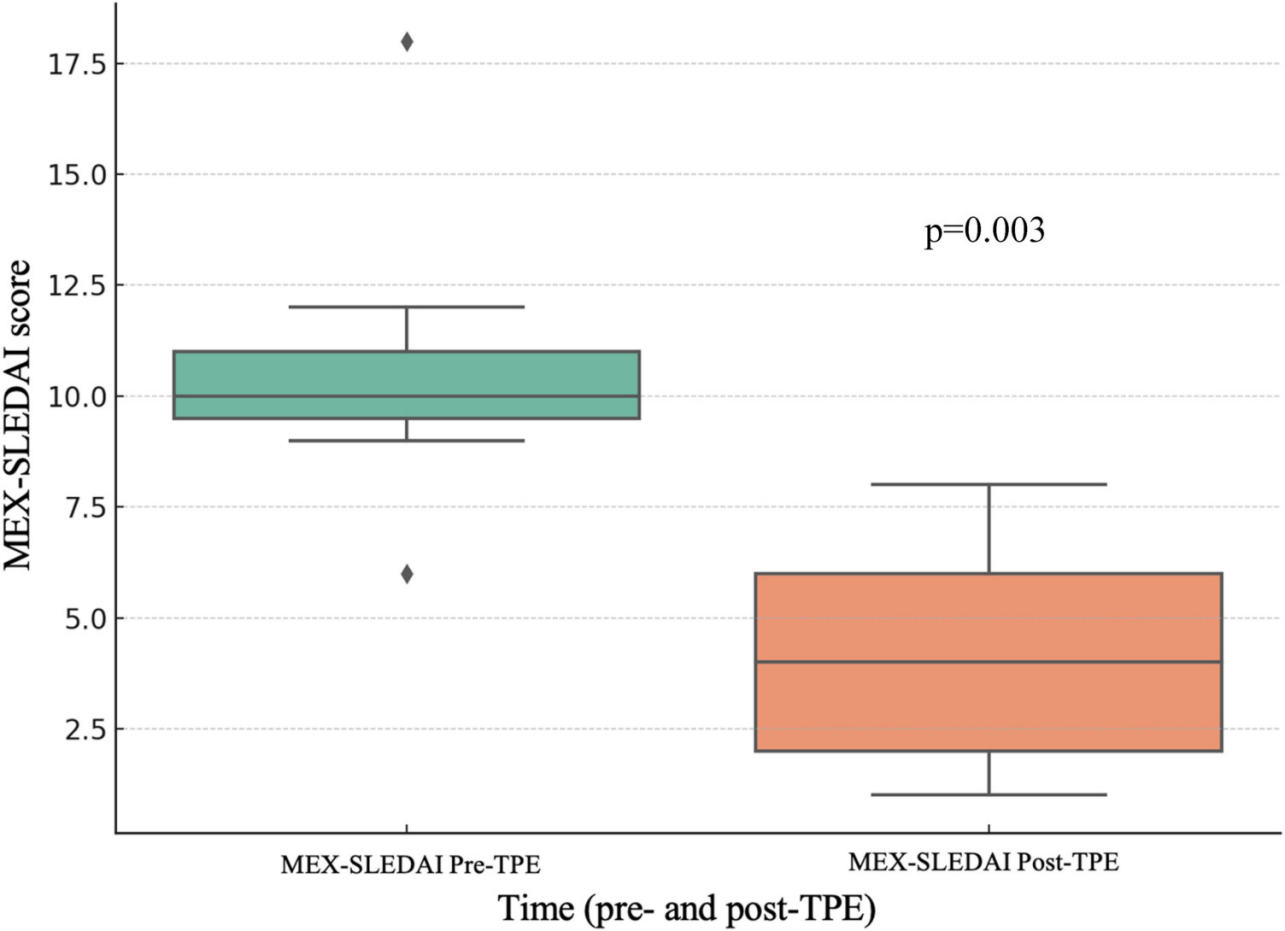
Disease activity progression (MEX-SLEDAI) in SLE patients without sepsis before and after TPE.

This study evaluated the experience of TPE in a cohort of pediatric patients with autoimmune diseases treated at the Hospital Infantil de México Federico Gómez. Demographic, clinical, laboratory variables, clinical response, and mortality were analyzed. In our cohort, the median age at diagnosis was 12 years (IQR 8–14), with a female predominance (68%). The most frequent diagnosis was SLE in 23 patients (56.1%). These findings are consistent with the literature, where SLE commonly manifests between 10 and 12 years of age, with a clear female predominance (14, 15). Some studies have also reported a median time to SLE diagnosis of 1.4 months in peri-pubertal patients and 1.0 month in adolescents (16), whereas in our cohort, TPE was performed after a median disease duration of 2 months, in the context of a severe refractory initial presentation. Several studies have demonstrated that patients with autoimmune diseases have an increased risk of developing sepsis, especially those treated with glucocorticoids or biologic agents, which increases morbidity and mortality risk (17). In our cohort, 39% of patients had a concomitant diagnosis of sepsis. A median of 5 TPE sessions per patient was performed. Albumin was the most commonly used replacement fluid (92.7%); however, fresh frozen plasma (FFP) was used in three patients due to the presence of sepsis and coagulation abnormalities, conditions that require plasma factor replacement. This choice aligns with recommendations from the ASFA guidelines, where the number of TPE sessions varies depending on the underlying disease (18). For systemic autoimmune diseases, 3–7 sessions are usually indicated, performed on consecutive or alternate days. According to the ASFA classification, the most frequent therapeutic indication corresponded to category III (48.8%), followed by category II (26.8%) and category I (24.4%). Regarding procedural safety, TPE is considered a low-complication intervention; the most frequently described adverse reactions include hypotension, hypocalcemia, nausea/vomiting, and urticaria (19). In our cohort, these events were rare but did occur, with palpitations being the most frequently reported effect, confirming that TPE is a well-tolerated therapy in the pediatric population. Before starting TPE, 82.5% of patients had hemodynamic compromise, followed by respiratory and neurological involvement (74.2% and 70.4%, respectively), consistent with studies where TPE has been used in patients with multiple organ failure or requiring advanced life support (20). Response rates to TPE vary according to the type of autoimmune disease involved (8, 13, 21). In our cohort, 85.4% of patients achieved partial or complete remission, while the remainder had persistent disease. Notably, 39.1% of patients with SLE and 83.3% with autoimmune encephalitis achieved complete remission. These findings are consistent with other series reporting favorable responses to TPE in patients with SLE and autoimmune encephalitis (9, 22). Conversely, the coexistence of SLE and sepsis was strongly associated with mortality, underscoring the importance of early recognition and aggressive management of infection in these cases. On the other hand, patients with MAS, primarily associated with sJIA, showed a limited response to treatment, which is consistent with previous reports suggesting a more severe clinical course and more variable response to TPE in this context (23, 24). In overlap syndromes, the response was heterogeneous, although two out of three patients achieved complete remission. Less frequent diseases—such as juvenile dermatomyositis (25), ANCA-associated vasculitis (6), Takayasu arteritis (26), APS (27), and Kawasaki disease (28)—were individual cases with variable responses. Given the limited representation of these subgroups, firm conclusions cannot be drawn; however, the findings suggest that TPE could be a useful therapeutic alternative in selected cases with refractory disease or severe systemic involvement. Mortality in our study was not directly associated with the TPE procedure but rather with severe systemic inflammatory response and shock status. The observed mortality rate was 14.6% (6 patients). Five of the six deceased patients (83.3%) had an SLE diagnosis, and all presented with septic shock. Comparing deceased and surviving patients, the presence of sepsis was significantly associated with mortality (*p* < 0.001). In some cases, septic shock was accompanied by severe complications such as disseminated intravascular coagulation (DIC), massive bleeding, or toxic shock syndrome (TSS). These findings align with a recent systematic review and meta-analysis, which found no significant difference in mortality between children with severe sepsis or septic shock who received TPE and those who did not (29). Fatal outcomes occurred in half of the patients by the end of the last TPE session, while the others showed progressive clinical deterioration. Regarding inflammatory biomarkers, deceased patients exhibited a pattern suggestive of more intense inflammation and potential immune dysfunction. Although differences did not reach statistical significance, these patients showed notably lower ESR levels (median 0 mm/h vs. 22 mm/h). This may relate to an exacerbated systemic inflammatory response leading to decreased fibrinogen — one of the main determinants of ESR — due to consumptive coagulopathy and reduced synthesis from hepatic dysfunction (30). Additionally, various studies have reported discordance between ESR and CRP values in hospitalized patients, attributed to multiple factors such as differences in cytokine stimulation, inherent variations in normalization processes, and false positive or negative characteristics of individual acute phase reactants (31). In contrast, higher levels of CRP, procalcitonin, and ferritin were observed, markers associated with systemic inflammation, sepsis, and cytokine release syndrome (32). In univariable analysis, longer disease duration was significantly associated with increased risk of death, suggesting that a more prolonged clinical course might reflect more aggressive, treatment-refractory disease or greater cumulative damage. This finding could be due to delayed diagnosis, insufficient response to conventional therapies, or sustained inflammatory burden, factors that may negatively influence prognosis (33, 34). However, this association lost statistical significance in multivariable analysis adjusted for age and sex, indicating that these factors might modulate or confound the relationship between disease duration and mortality. Finally, in SLE patients without sepsis, disease activity was assessed using the MEX-SLEDAI score before and after TPE. Before TPE, all patients had moderate to very severe activity (scores 6–18). After treatment, MEX-SLEDAI scores decreased in all cases, reaching inactive or mild activity in 7 patients (63.6%) and moderate activity in 4 patients (36.4%). These findings suggest significant clinical improvement following TPE in this sepsis-free subpopulation, consistent with observations in other series (9) (see Figure 1). Limitations of our study include its cross-sectional design and single-center setting. The small sample size also limits generalizability and statistical power. Furthermore, the heterogeneity of autoimmune diseases included makes comparison between diagnostic subgroups challenging, as each entity presents distinct pathophysiological mechanisms, activity levels, clinical evolution, and therapeutic responses. This variability may influence TPE efficacy, clinical presentation at the time of the procedure, and outcomes such as remission or mortality. Therefore, overall results should be interpreted with caution, as they may not be generalizable to each specific disease. Nevertheless, this is one of the largest reports from a pediatric referral center in Latin America. Multicenter studies with larger sample sizes are needed to confirm these findings in the pediatric population.

## Conclusion

TPE was safe in pediatric patients with severe autoimmune diseases, showing adequate clinical response and a low frequency of complications. Disease duration was the main factor associated with mortality, and deceased patients exhibited a more severe inflammatory profile, without the procedure itself increasing the risk of death. Notably, mortality occurred exclusively in patients with sepsis, and the majority had underlying SLE, underscoring the vulnerability of this subgroup when infection is present. Early use of TPE in patients with severe, refractory autoimmune diseases—particularly those with SLE without sepsis and autoimmune encephalitis—showed favorable clinical outcomes. Our findings support the use of TPE as a valuable therapeutic option in refractory or critically presenting cases, particularly in diseases such as SLE or autoimmune encephalitis, but also in other autoimmune conditions including overlap syndromes, MAS, juvenile dermatomyositis, APS, ANCA-associated vasculitis, Takayasu arteritis, and Kawasaki disease. This study provides novel insights that may guide clinical decision-making in pediatric contexts requiring rapid intervention for organ failure or severe systemic inflammation. Additionally, our results highlight the need to standardize criteria for initiating TPE in children and to establish disease-specific protocols. This is one of the largest studies conducted at a Latin American center and the first to report data on TPE use in an exclusively Mexican pediatric cohort. Prospective multicenter studies are needed to validate these findings, better define patient profiles that most benefit from the procedure, and explore predictive biomarkers of response or complications.

## Data Availability

The raw data supporting the conclusions of this article will be made available by the authors, without undue reservation.
