# Supplementary material for: Therapeutic plasma exchange in autoimmune diseases: a retrospective study in a tertiary pediatric hospital in Mexico
Source: Front Pediatr. 2025 Oct 24;13:1680460. doi: 10.3389/fped.2025.1680460 (PMC12592088; doi:10.3389/fped.2025.1680460)
Supplement: Supplementary file 1 [file Supplementaryfile1.docx]

**Supplementary Material 1.** The procedures were performed via a central venous catheter using the Spectra Optia® apheresis system by TERUMO BCT, which employs continuous flow centrifugation and optical detection (Automated Interface Management, AIM) for blood component separation. In patients weighing more than 20 kg, total blood volume (TBV) was automatically calculated using Nadler’s formula*, which considers sex, weight, height, and hematocrit, using the following expressions: for males, TBV = (0.3669 × height³) + (0.03219 × weight) + 0.6041; and for females, TBV = (0.3561 × height³) + (0.03308 × weight) + 0.1833. In patients weighing less than 20 kg, TBV was manually estimated using Gilcher’s rule***. Plasma volume (PV) was calculated using Kaplan’s formula**: PV = TBV × (1 – Hct), and the exchange volume corresponded to 1.5 blood volumes, according to ASFA recommendations. The most commonly used replacement fluid was 5% albumin, prepared under laminar flow hood by diluting 25% albumin with 0.9% saline to ensure an isovolumetric and iso-oncotic exchange while maintaining the patient’s oncotic pressure. ACD-A solution (acid citrate dextrose, formula A) was used as anticoagulant. To prevent citrate-induced hypocalcemia, calcium gluconate (100–200 mg/kg) was routinely added to the replacement solution. Blood flow was adjusted between 25 and 50 mL/min depending on the patient’s clinical condition, procedural tolerance, and extracorporeal circuit volume.

***Nadler formula**

Males

TBV = (0.3669 × height³) + (0.03219 × weight) + 0.6041

Females

TBV = (0.3561 × height³) + (0.03308 × weight) + 0.1833

TBV, Total blood volume.

****Kaplan's rule**

TBV × (1 − HCT/100) = PV

PV, Plasma volume.

*****Gilcher's rule in pediatrics**

10 kg: 80 mL/kg

10 to 20 kg: 77 mL/kg

20 kg: 70 mL/kg

**Table 4, supplementary material 2**. Disease activity measured by MEX-SLEDAI before and after therapeutic plasma exchange (TPE) in SLE patients without sepsis (n = 11). A score of 0–1 was considered inactive, 2–5 mild activity, 6–9 moderate activity, 10–13 severe activity, and >14 very severe activity. The median pre-TPE MEX-SLEDAI score was 10 (interquartile range [IQR] 9–11), and the median post-TPE score was 4 (IQR 2–6). Disease activity before and after TPE was compared using the Wilcoxon signed-rank test, showing a statistically significant reduction in disease activity (median pre-TPE 10 [IQR 9–11] vs. median post-TPE 4 [IQR 2–6], p = 0.0030).

| **Patient** | **MEX-SLEDAI Pre-TPE** | **MEX-SLEDAI Post-TPE** |
| --- | --- | --- |
| 1 | 18 | 5 |
| 2 | 10 | 2 |
| 3 | 6 | 4 |
| 4 | 10 | 6 |
| 5 | 11 | 8 |
| 6 | 12 | 6 |
| 7 | 10 | 6 |
| 8 | 9 | 1 |
| 9 | 10 | 2 |
| 10 | 9 | 1 |
| 11 | 11 | 3 |
